# Assessing the impact of Ascariasis and Trichuriasis on weight gain using a porcine model

**DOI:** 10.1371/journal.pntd.0010709

**Published:** 2022-08-19

**Authors:** Bradley Whitehead, Stig M. Thamsborg, Matthew J. Denwood, Peter Nejsum

**Affiliations:** 1 Department of Clinical Medicine, Aarhus University, Aarhus, Denmark; 2 Department of Infectious Diseases, Aarhus University Hospital, Aarhus, Denmark; 3 Department of Veterinary and Animal Sciences, University of Copenhagen, Copenhagen, Denmark; Istituto Superiore di Sanità, ITALY

## Abstract

**Background:**

Infections with *Ascaris lumbricoides* and *Trichuris trichiura* remain significant contributors to the global burden of neglected tropical diseases. Infection may in particular affect child development as they are more likely to be infected with *T*. *trichiura* and/or *A*. *lumbricoides* and to carry higher worm burdens than adults. Whilst the impact of heavy infections are clear, the effects of moderate infection intensities on the growth and development of children remain elusive. Field studies are confounded by a lack of knowledge of infection history, nutritional status, presence of co-infections and levels of exposure to infective eggs. Therefore, animal models are required. Given the physiological similarities between humans and pigs but also between the helminths that infect them; *A*. *suum* and *T*. *suis*, growing pigs provide an excellent model to investigate the direct effects of *Ascaris* spp. and *Trichuris* spp. on weight gain.

**Methods and results:**

We employed a trickle infection protocol to mimic natural co-infection to assess the effect of infection intensity, determined by worm count (*A*. *suum*) or eggs per gram of faeces (*A*. *suum* and *T*. *suis*), on weight gain in a large pig population (n = 195) with variable genetic susceptibility. Pig body weights were assessed over 14 weeks. Using a post-hoc statistical approach, we found a negative association between weight gain and *T. suis* infection. For *A. suum*, this association was not significant after adjusting for other covariates in a multivariable analysis. Estimates from generalized linear mixed effects models indicated that a 1 kg increase in weight gain was associated with 4.4% (p = 0.00217) decrease in *T*. *suis* EPG and a 2.8% (p = 0.02297) or 2.2% (p = 0.0488) decrease in *A*. *suum* EPG or burden, respectively.

**Conclusions:**

Overall this study has demonstrated a negative association between STH and weight gain in growing pigs but also that *T*. *suis* infection may be more detrimental that *A*. *suum* on growth.

## Author summary

Infections with the roundworm, *Ascaris lumbricoides* and the whipworm *Trichuris trichiura* are estimated to affect over 800 and 400 million people, respectively. Infections are most common in children and whilst very heavy infections present clear pathology it is less clear what effect more moderate infection have on the growth and development of children. Attempts to quantify the detrimental effects in humans have been inconclusive to date due to multiple confounding variables. Therefore, animal models are required. Pigs are natural hosts to two very closely related helminths; *A*. *suum* and *T*. *suis* and therefore we have investigated the effects of infection on growing pigs. This study has identified an association of infection levels with both helminths with reduced weight gain but also that whipworm infection may be more detrimental to weight gain than giant roundworm infection. Given the overall high prevalence of *A*. *lumbricoides* and difficulties in successfully clearing whipworm infections (multiple doses of anthelminthic are required), this work highlights the importance of drug administration programs targeted to clear these infections in children.

## Introduction

*Ascaris lumbricoides* and *Trichuris trichiura* are two of the most prevalent soil-transmitted helminths (STH), and despite extensive mass drug administration (MDA) programs these helminths remain a significant contributor to the burden of neglected tropical diseases [1].

Whilst the impact of acute pathology is clear, for example *Trichuris* dysentery syndrome or intestinal blockage resulting from heavy infections of *T*. *trichiura* or *A*. *lumbricoides*, respectively [2], the effect of moderate infection intensity has proven difficult to quantify [2]. Children are more likely to be infected with *T*. *trichiura* and/or *A*. *lumbricoides* and to carry higher worm burdens than adults, suggesting that infection has the most impact on child development in endemic areas [2]. However, data describing the impact of *A*. *lumbricoides* or *T*. *trichiura* on the growth and development of infected children are inconclusive [3,4]. The difficulty of determining the association of infection intensity and development in observational field studies is confounded by a lack of knowledge of; infection history, nutritional status, presence of co-infections and levels of exposure to infective eggs. Therefore, animal models are required to determine the direct and continuous effect of *Ascaris* spp. and *Trichuris* spp. on weight gain. Pigs are physiologically similar to humans, particularly in gastrointestinal structure and function, and are infected by two closely related helminths; *A*. *suum* and *T*. *suis* [5–9]. *A*. *suum* and *A*. *lumbricoides* are genetically very similar, to the extent that debate persists if they are indeed one species [5,10]. Likewise, *T*. *suis* is a sibling species to *T*. *trichiura* and bears a closer relationship than to whipworms of ruminants. Importantly, both these porcine helminths are capable of infecting humans [11]. As such, pig models have been crucial to our understanding of *Ascaris* spp. and *Trichuris* spp. infection pathophysiology and provide a highly suitable large mammal model to investigate the effects of STH infection intensity on weight gain, particularly during the early growth phase when immunity builds up [12]. However, the direct effects of parasitism on pig growth have also been difficult to determine under normal production conditions with studies influenced by multiple variables, including: individual host susceptibility related to genetics [13] and nutritional status, parasite species, levels and timing of exposure, presence of co-infection, treatment regimens and feed composition [14]. Our previous studies have identified that 32–73% of the phenotypic variance observed in *T*. *suis* infection was related to genetics whilst this was 29–45% for *A*. *suum* [13]. A meta-analysis of available literature encompassing 16 studies of pigs parasitized with helminths, of which 13 included *A*. *suum* and/or *T*. *suis* infection, did demonstrate that the presence of helminths in growing pigs negatively impacted feed intake, feed-conversion rate and weight gain [15]. However, infection intensity was not assessed; therefore, the impact of worm burden upon weight gain remains unknown. We predict helminth infection is detrimental to weight gain in growing pigs and aim to determine the relationship between worm burden and weight gain. Therefore, we have employed a trickle infection scheme to mimic natural co-infection with a controlled but constant exposure to assess the effect of infection intensity, determined by worm count (*A*. *suum*) or eggs per gram of faeces (*A*. *suum* and *T*. *suis*), on weight gain in a large pig population (n = 195) with variable genetic susceptibility [13]. Pig body weights and faecal egg counts for *T*. *suis* and *A*. *suum* were assessed over 14 weeks as well as macroscopic *A*. *suum* worm counts upon sacrifice. Thereby enabling an accurate determination of infection intensity throughout the 14 weeks increasing the accuracy and power of this study compared to that possible in field studies in humans.

## Materials and methods

### Ethics statement

The experimental study was approved by the Experimental Animal Unit, University of Copenhagen according to Federation of European Laboratory Animal Science Associations (FELASA) guidelines and recommendations and performed under the Danish experimental animal licence no. 2000/561-321, Ministry of Food, Agriculture and Fisheries of Denmark. The study was performed prior to the establishment of a research ethics committee (institutional review board) at University of Copenhagen in 2016.

### Study design

The study was performed as previously described [13]. Briefly, 195 piglets were obtained from Danish Landrace-Yorkshire sows sired by Duroc boars from two farms in two farrowing rounds. At 8 weeks of age, pigs were stratified according to sex, litter and farm of origin then randomly allocated to six helminth-free paddocks. Pigs were in the paddocks for 2 weeks prior to infection with *A*. *suum* and *T*. *suis* eggs at a rate of 25 and 5 eggs/kg body weight/day, respectively, given twice weekly in feed. Dosing was adjusted weekly according to mean pig body/live weight per paddock and levels according to previous studies [16]. The feed was a standard diet consisting of ground barley supplemented with a commercial mixture of protein and minerals and pigs had free access to water. Faecal egg counts were taken for each pig at weeks 0, 6, 7, 8, 9, 10, 12 and 14 post first infection (P.I), using a modified McMaster protocol [13] and reported as eggs per gram (EPG). Pig live weights were recorded at 0, 7 and 14 weeks P.I. Pigs were sacrificed at week 14 P.I, and the number of macroscopic *A*. *suum* (more than 1 cm) was counted after opening the small intestine and sieving of the contents. As very few pigs were coprologically positive for *T*. *suis* at week 14 P.I., their worm counts were not determined.

### Post-hoc pairing procedure

An unavoidable consequence of the study design was clustering of the 195 piglets within dam (n = 19), sire (n = 13), sex (n = 2), and farm (n = 2). In order to avoid having to control for these variables in the primary statistical analysis, a post-hoc pairing procedure was used to ensure that these variables were perfectly balanced with respect to the datasets to be analysed resulting with n = 174. The procedure used was as follows:

- Divide the animals into subsets representing each of the 38 combinations of dam/sire/sex/farm observed in the data, i.e. each subset was composed of same-sex full siblings.
- For each subset, rank the animals by decreasing weight gain either (1) between weeks 0–7, (2) between weeks 7–14, or (3) between weeks 0–14. Ties were broken by ranking on piglet ID (i.e. pseudo-randomly).
- For the 21 subsets with odd numbers of piglets, discard the middle-ranked piglet so that all subsets have an even number of observations. Divide the remaining ranked piglets into weight gain groups (high and low) so that each of these groups ended with the same number of animals.
- Pair the piglets within each subset by matching the 1^st^ ranked high weight gain piglet with 1^st^ ranked low weight gain piglet, then the 2^nd^ ranked in each weight group, and so on. This generates a total of 87 pairs, with 9 subsets contributing a single pair, 13 subsets contributing two pairs, 13 subsets contributing three pairs, 2 subsets contributing four pairs, and 1 subset contributing five pairs.
- Generate a pair ID incorporating the subset number and pair number, and weight group (low weight gain or high weight gain), for each piglet in the dataset

Based on this procedure we generated three paired datasets, with each representing pairing based on weight gain over the different periods (and therefore based on different piglets). Note that the paddock number was not used to inform the pairing procedure as allocation to paddocks was performed randomly.

For each of the three paired datasets, comparisons were then made between weight groups for:

- *T*. *suis* EPG at weeks 6, 7, 8, 10, 12, 14
- Overall mean *T*. *suis* EPG
- *A*. *suum* EPG at weeks 6, 7, 8, 10, 12, 14
- Overall mean *A*. *suum* EPG
- *A*. *suum* worm count at post mortem

The mean estimated difference between weight groups was calculated based on the mean of the intra-pair differences, with statistical significance assessed using a paired Wilcoxon signed rank test of the difference being symmetric about zero. Due to the large number of statistical comparisons entailed with comparing EPG at 6 time points, mean EPG and worm count (for *A*. *suum*), the resultant p-values were adjusted using Holm’s method to correct for multiple analyses [17]. This correction was performed separately for each of the six combinations of paired dataset and parasite species. Empirical cumulative distribution function (ECDF) plots were produced to visualise the distribution of each of these outcomes between weight groups, with log10 transformation of the over-dispersed count data (after adding a constant of 1 to all values) used to aid visualisation.

### Statistical modelling

In order to support the more fine-grained evidence provided by the paired data analysis, an estimate of the overall magnitude of the association between weight gain and parasite burden was obtained using a generalized linear mixed effects model. For this procedure, the total EPG for each parasite was summed within the same animal over the entire time-period to produce a single animal-level observation for each (n = 195). Along with the *A*. *suum* worm count, these were then used as the response variable using a generalized linear mixed effects model (GLMM) with log link and negative binomial response. A linear effect of weight gain for the same animal (over the entire time period) was used as the primary explanatory variable of interest, and random effects of pen and combined dam/sire/sex/farm were included to control for these two sources of clustering. The same model was fitted to each of the three datasets separately, and estimates of the magnitude and significance of the association with weight gain were extracted assuming that the relationship is linear in nature.

In order to assess the validity of the assumption of linearity, the three analyses described above were repeated using a generalized additive model (GAM) in place of the GLMM, with a thin plate regression spline used for the effect of weight gain. Random effects of pen and combined dam/sire/sex/farm were included as before. These models were used to generate estimates and 95% confidence intervals for the (potentially non-linear) relationship between weight gain and EPG/worm count (on the log scale). Graphical representations of these estimated relationships were produced for visualization, with the y axis scaled to represent the mean EPG rather than sum of counts, and actual data observations overlaid for comparison.

In order to assess the potential for confounding between the two parasite species, a linear mixed effects model was fit to the weight gain for each animal with explanatory variables of log-transformed mean *A*. *suum* EPG (+1) and log-transformed mean *T*. *suis* EPG (+1), as well as random effects of pen and combined dam/sire/sex/farm as above. Confounding was tested by comparing the estimates from the multivariable model to estimates for each of the parasites in turn, and a likelihood ratio test was used on the interaction between the parasites in the multivariable model.

All statistical analyses were two-sided (alpha = 0.05) and were performed using R version 4.1.2 [18] with LMM and GLMM provided by the “lme4” package [19] and GAM provided by the “mgcv” package [20].

## Results

**Fig 1:**
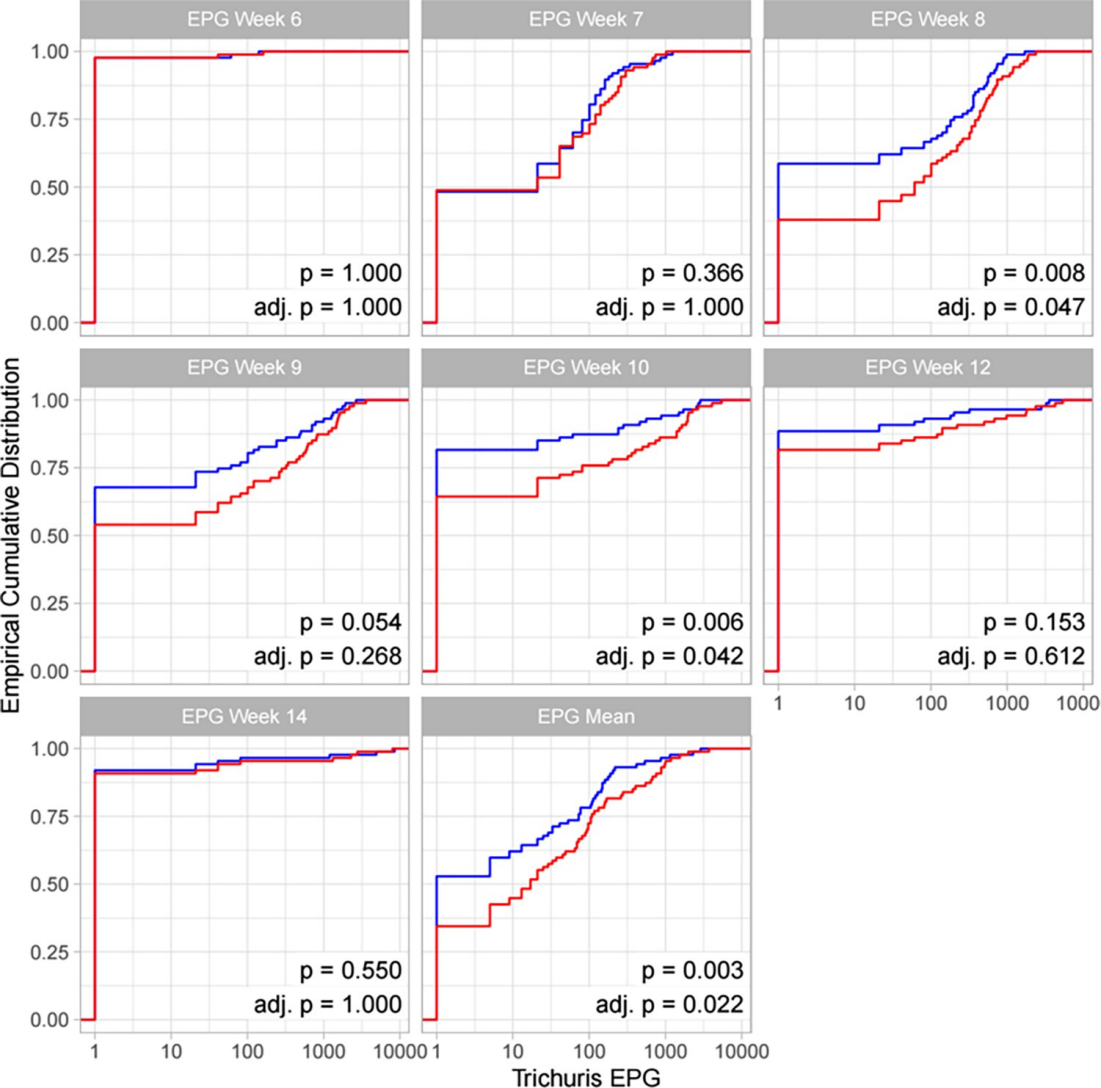
Empirical cumulative distribution function plots of *Trichuris suis* EPG at each time point (EPG week 6–14) and mean EPG throughout time course (EPG mean) for low weight gain animals (red) compared to high weight gain animals (blue) grouped by pig weight gains at weeks 0–14 (n = 174). p-values based on Wilcoxon signed rank tests are given in addition to the same p-values adjusted according to Holm’s method (Holm, 1979) [17].

**Fig 2:**
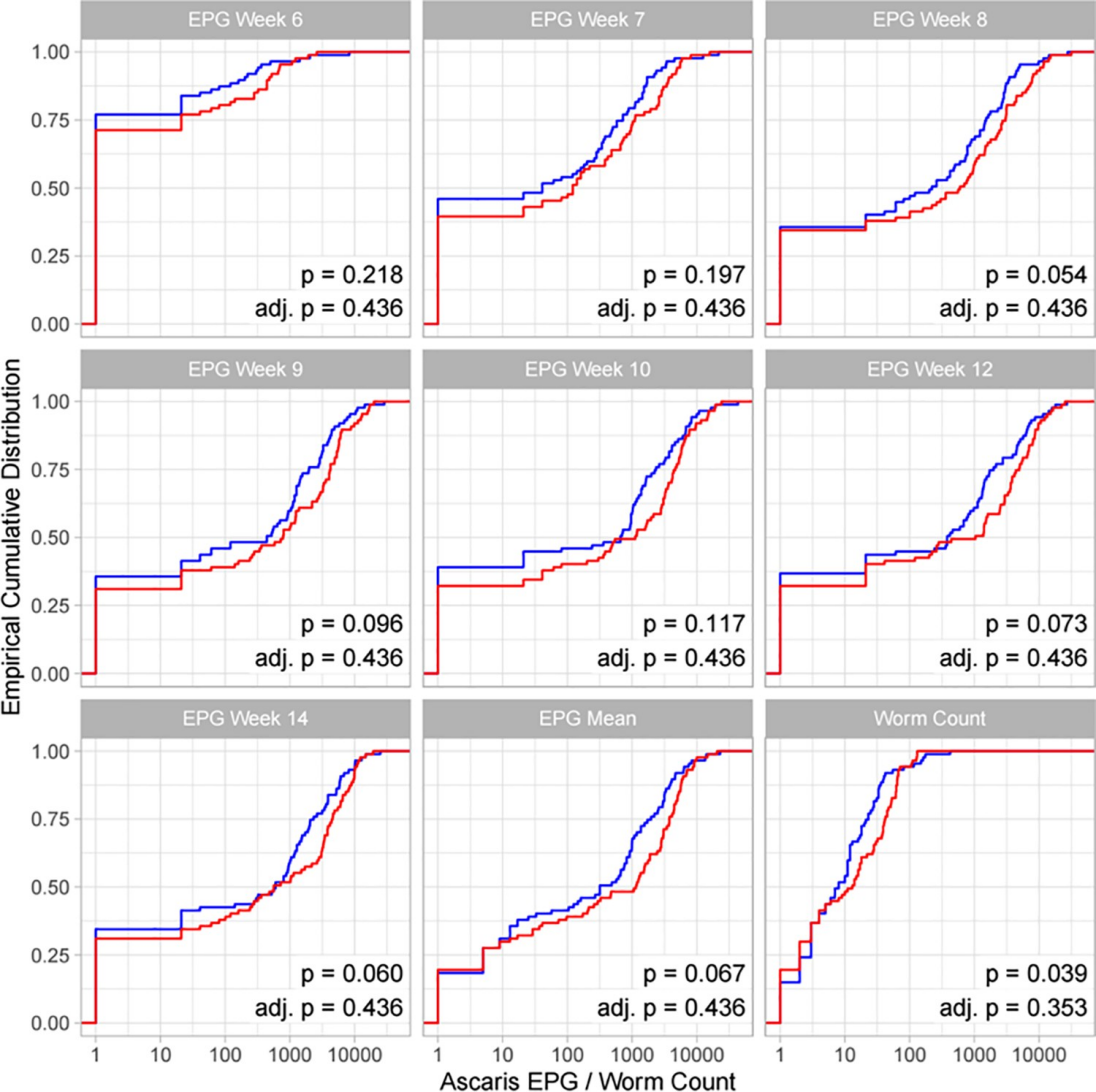
Empirical cumulative distribution function plots of *Ascaris suum* EPG at each time point (EPG week 6–14) and mean EPG throughout time course (EPG mean) for low weight gain animals (red) compared to high weight gain animals (blue) grouped by pig weight gains at weeks 0–14 (n = 174). p-values based on Wilcoxon signed rank tests are given in addition to the same p-values adjusted according to Holm’s method (Holm, 1979) [17].

The percentage of pigs with positive egg counts at the peak of infection were >60% for *A*. *suum* and just under 50% for *T*. *suis*[13]. Empirical cumulative distribution function (ECDF) plots showing EPG and worm count (*A*. *suum* only) at each time point and for each of the weight gain groups are shown for *T*. *suis* (Fig 1) and *A*. *suum* (Fig 2). Each plot also displays difference in EPG/worm count (low weight gain minus high weight gain) and (adjusted) p-value for the comparison shown (Figs 1 and 2).

Significant differences in the distribution of *T*. *suis* EPG between low and high weight gain (0-14WG) groups were shown at week 8 (adj.p = 0.047), week 10 (adj.p = 0.042) and throughout the study covering weeks 0–14 (adj.p = 0.022) (Fig 1). However, grouping of pigs based on weight gains at 0–7 weeks showed no significant association between EPG and weight gain at any time point (S1 Fig). Conversely, when pigs were grouped according to weight gain in weeks 7–14, weight gain was again significantly associated with EPG in weeks 7, 8, 9, 10, and over the 14-week period (adj.p = 0.016) (S2 Fig). This is also reflected in the difference in EPG between groups (difference between the red and blue lines), which was highest at weeks 8, 9, and 10 for weight gain over the period week 0–7. The estimated mean difference was positive (i.e. higher for low weight gain compared to high weight gain groups, so that the red line sits below/to the right of the blue line) for all comparisons except at week 14.

For *A*. *suum* there was an indication that worm count at week 14 was associated with lower weight gain when grouped using pig weight gains from 0–14 weeks (Fig 2). However, this result was not significant after adjustment for multiple tests (Fig 2). Likewise, no significant association was observed for *A*. *suum* and weight gain when groups were selected based on pig weights at week 0–7 (S3 Fig) or week 7–14 (S4 Fig).

No significant differences between the distribution of EPG were observed for *T*. *suis* or *A*. *suum* when groupings were performed according to pig live weight at week 0 (S5 and S6 Figs).

**Fig 3:**
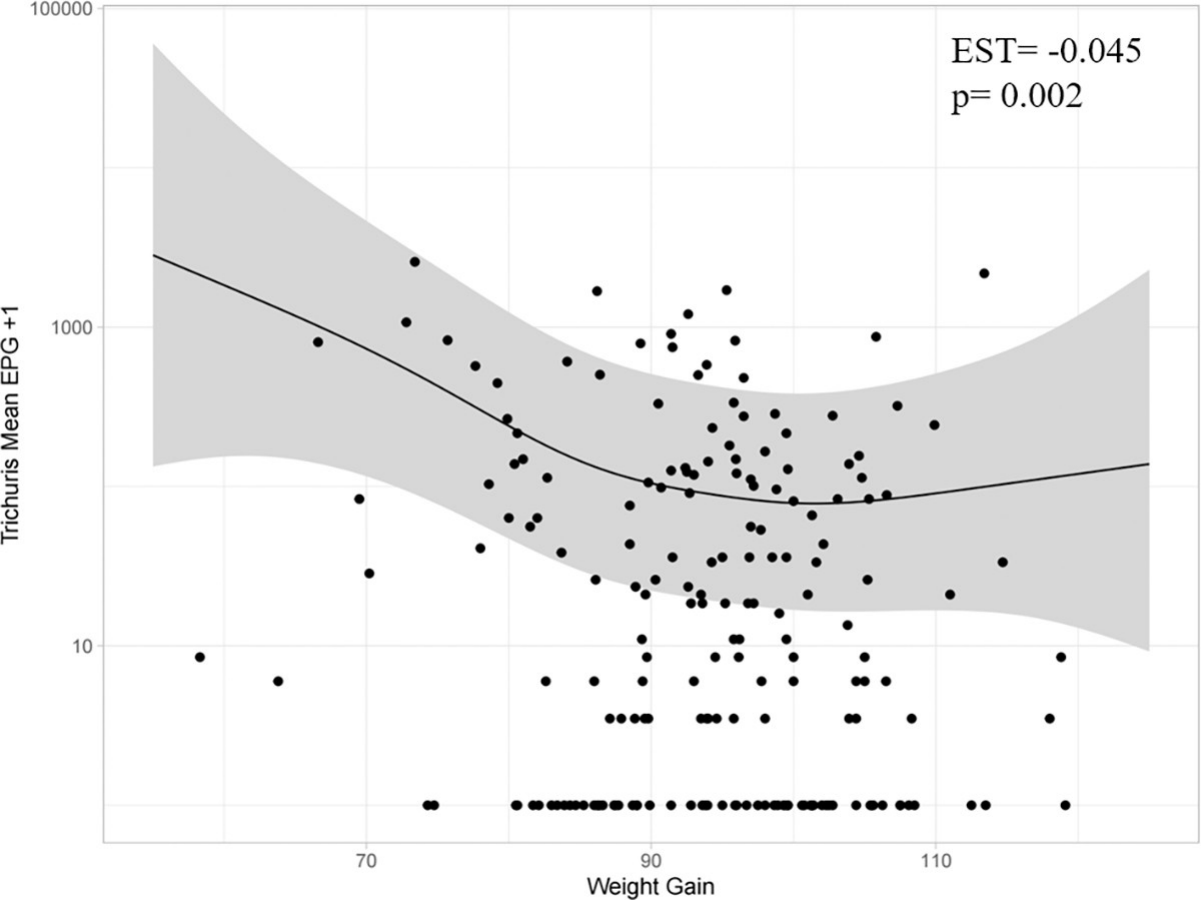
The relationship between *Trichuris suis* mean EPG (log scale) and weight gain (week 0–14) estimated using a generalised additive model (GAM) (n = 195). The estimate is shown by solid black line and 95% CI are shown in grey shading. Individual data points have been overlaid for reference (black dots), with a constant of 1 added before log transformation for the purposes of visualization only. Inset are the estimate (EST) and p-value generated using a generalised linear mixed model (GLMM) assuming that the relationship is linear.

**Fig 4:**
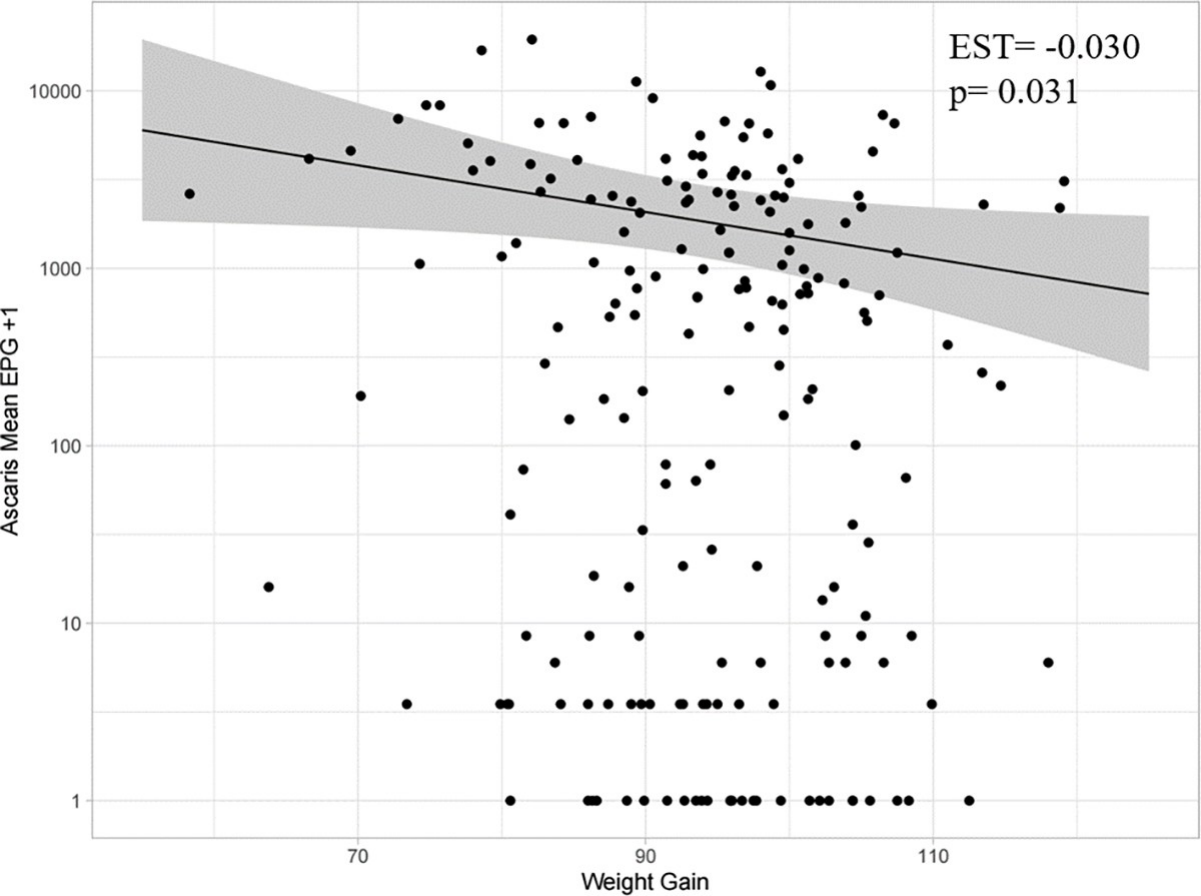
The relationship between *Ascaris suum* mean EPG (log scale) and weight gain (week 0–14) estimated using a generalised additive model (GAM) (n = 195). The estimate is shown by solid black line and 95% CI are shown in grey shading. Individual data points have been overlaid for reference (black dots), with a constant of 1 added before log transformation for the purposes of visualization only. Inset are the estimate (EST) and p-value generated using a generalised linear mixed model (GLMM) assuming that the relationship is linear.

**Fig 5:**
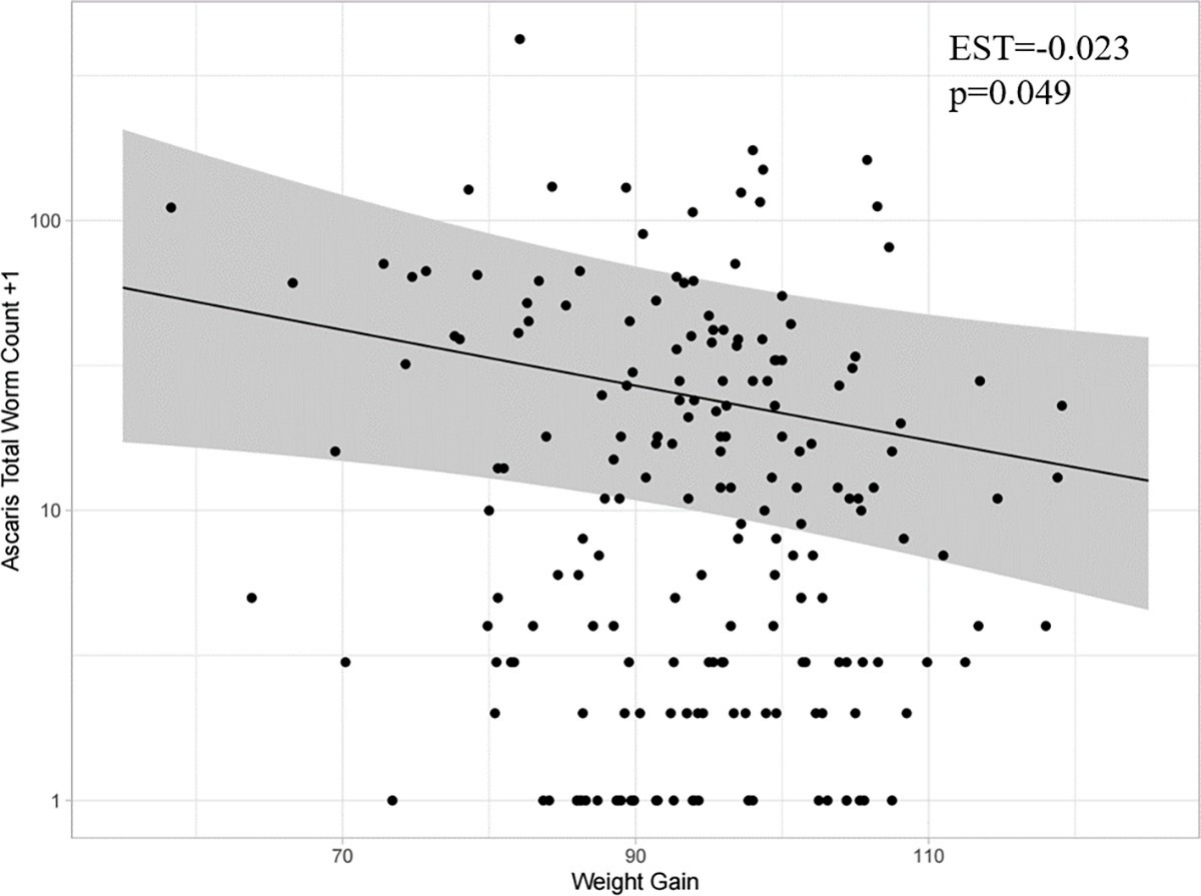
The relationship between *Ascaris suum* total worm counts (log scale) and weight gain (week 0–14) estimated using a generalised additive model (GAM) (n = 195). The estimate is shown by solid black line and 95% CI are shown in grey shading. Individual data points have been overlaid for reference (black dots), with a constant of 1 added before log transformation for the purposes of visualization only. Inset are the estimate (EST) and p-value generated using a generalised linear mixed model (GLMM) assuming that the relationship is linear.

To examine the effect of parasite load on weight gain (week 0–14) using EPG or worm count as a proxy for helminth burden, we first generated a general additive model (GAM) to determine if the relationship between weight gain and the expected EPG/worm count (on the log scale) is linear (Figs 3, 4 and 5). There was no evidence for a non-linear relationship between weight gain and either *A*. *suum* EPG (Fig 4) or worm count (Fig 5), with the GAM producing an estimated degrees of freedom (EDF) of ~1 when fit to the counts using a negative binomial distribution with log link. However, the relationship between *T*. *suis* EPG and weight gain was not completely linear (EDF = 2.8) due to the plateau observed between moderate and higher weight gains (Fig 3).

Despite this caveat for *T*. *suis*, we proceeded to apply a generalised linear mixed model (GLMM) to provide a quantification for the relationship between helminth burden and weight gain (week 0–14) in our 195 pigs. The results can be summarised as follows:

- A 1 kg increase in weight gain was associated with a 4.4% (p = 0.00217) decrease in *T*. *suis* EPG
- A 1 kg increase in weight gain was associated with a 2.8% (p = 0.02297) decrease in *A*. *suum* EPG
- A 1 kg increase in weight gain was associated with a 2.2% (p = 0.0488) decrease in *A*. *suum* burden, as determined by worm count

No confounding effects or interactions between *T*. *suis* and *A*. *suum* were observed: coefficient estimates were similar between models when fit singly or in combination (-1.8848 vs -1.7938 for *T*. *suis*, -0.6473 vs -0.5078 for *A*. *suum*) and there was no evidence for an interaction (p = 0.961).

## Discussion

The overall impact of STH infection on weight in humans and some animals has been difficult to determine due to complication by multiple confounders, not least the individual susceptibility of hosts to infection and resultant aggregation of worm burden across the host population. Likewise, nutritional status can affect outcomes. For example, it has been shown that the effects of helminth infection on pig weight gain are exacerbated by low protein and low iron diets [21,22]. This is of relevance to areas with restricted dietary conditions in the developing world, but the feed composition selected for this study was protein replete to enable the investigation of the direct pathological effects of infection intensity on weight gain. Therefore, this study aimed to determine if worm burden was negatively associated with weight gain in growing pigs raised on a standard commercial feeding and exposed to trickle infection to mimic natural infection. *A*. *suum* and *T*. *suis* were selected as relevant helminth infections due to the overall prevalence of their sibling parasites in humans, *A*. *lumbricoides* and *T*. *trichiura*. Importantly, the two helminths selected for this study produce environmentally resistant eggs, making control problematic. For example, infectivity increases two years after environmental contamination and viable *A*. *suum* eggs can be present up to 13 years later [23]. Likewise, *T*. *suis* has been reported to survive for 5 years [24] and 11 years [25] in the environment. Therefore, the potential for reinfection in periods between mass drug administration programs remains high.

In this study *A*. *suum* worm load, as estimated by EPG, was not significantly associated with low weight gain but low weight gain groups displayed a tendency to possess higher macroscopic worm counts (burdens) compared to high weight gain groups, although this was not significant after correction for multiple statistical tests. Using an alternative statistical approach, we were able to estimate the effect of worm burden (EPG or worm count) on weight gain. Estimates from statistical models showed that a 1 kg increase in weight gain was associated with a 2.8% or 2.2% decrease in *A*. *suum* EPG or worm count, respectively, which we interpret biologically as an effect of increasing *A*. *suum* infection levels on decreasing weight gain. However, our analyses do not allow us to conclude that this relationship is causal.

The effect of *A*.*suum* infection intensity on weight gain is therefore detrimental, although this effect may not be substantial in well-nourished individuals. In addition, as we did not have an uninfected control group and feed intakes were not assessed, the full impact of *A*. *suum* exposure cannot be evaluated.

Of note, *T*. *suis* burden was associated with low weight gain in growing pigs throughout the 14 week study period based upon grouping by weight gain of sibling pairs in weeks 0–14. Interestingly, the estimated difference in EPG when grouping according to low and high weight gain in weeks 0–7 were generally small, but grouping by weight gain in week 7–14 showed a larger and statistically significant difference. With the caveat that weight gain groups are differently composed (rankings may be different in different periods), this suggests that most drastic effects on weight were mediated from weeks 7–14 by *T*. *suis* infection. This is likely the result of the infection dynamics of *T*. *suis* where it has been shown that most pigs have patent infections by week 7 P.I [26] and week 8 P.I [27] followed by expulsion of worms from most of the pigs. In hosts predisposed to heavier infections and unable to efficiently expel *T*. *suis*, the effects upon weight gain are likely aggravated and may explain why worm burden was significantly associated with low weight gain in these later weeks. This observation is consistent with that previously described in pigs infected with a moderate (1100 eggs/kg) or high (1650 eggs/kg) single dose of *T*. *suis* ova, where infection was associated with decreases in daily weight gain compared to low dose (550 eggs/kg) and non-infected pigs [28]. Notably, experimental mono-infection of pigs with *A*. *suum* did not negatively affect weight gain [29]. However accidental co-infection resulting from *T*. *suis* ova contaminated paddocks, did reduce weight gain, despite a comparable *A*. *suum* burden between mono and co-infected pigs at slaughter [30]. Conversely, a previous study investigating the effects of *T*. *suis* infection found no effects of infection upon weight gain in pigs infected with nil (0), low (400), medium (4000) or high doses (40,000) [31]. However, there were no significant differences in worm burden between dosing groups at slaughter so it is not possible to ascertain the effects of worm burden on weight gain. Likewise, no effects on weight gain were observed in *T*. *suis* infected pigs fed normal diet but low protein diet pigs did show reductions in productivity. In this study it was noted that worm burdens were aggregated in normal diet pigs but was more dispersed across the host population in low protein diet fed pigs which may obscure effects of *T*. *suis* infection on weight gain in normal diet pigs [22]. Combined, this is supportive of our study design with post-hoc grouping of pigs based upon weight gain in a given period in contrast to modulating worm burden through egg dosing regimens.

In the absence of weight gain data from uninfected pigs, we are unable to describe more than an association of worm burden with lower weight gain in this study and thus not a direct causal relationship. We can therefore not exclude that pigs that grow slower are more susceptible to worm infection. However, there seemed to be no association between the live weight before infection at week 0 P.I (approx. 10 weeks of age) and *A*. *suum* and *T*. *suis* worm loads (S5 and S6 Figs).

In conclusion, this study has identified a significant association between low weight gain and *T*. *suis* burden in co-infected growing pigs and a tentative association was observed for *A*. *suum*. Importantly, we found no interaction or confounding between the two parasites, indicating that the effect of *T*. *suis* burden is independent of *A*. *suum* infection intensity. Estimation of effects suggests that an increase in weight gain of 1kg is associated with a 4.4% decrease in *T*. *suis* EPG, which was reduced to 2.8% or 2.2% for *A*. *suum* EPG or worm count, respectively, suggesting that high *T*. *suis* infection levels result in greater potential losses in weight gain than *A*. *suum*. The pigs used in our study were fed a high quality protein replete diet and it is likely that the negative effects of *Ascaris* infections on malnourished pigs are exacerbated and this warrants investigation using a similar model to that presented here.

Whilst animal models are not directly translatable to human infections the similarities in pig physiology and the similarity in the helminths used in this study, that are capable of infecting humans, suggests these results are of importance to the development of children in endemic areas. Our demonstration of a negative association of *A*. *suum* infection with weight gain coupled with the high prevalence of *A*. *lumbricoides* suggest this STH remains an important yet neglected tropical disease. Likewise, the reduced impact of MDA on *T*. *trichiura* infection in endemic areas suggests the detrimental effect of *T*. *trichiura* is likely exacerbated and requires further investigation and the development of more effective anthelminthics targeting whipworm [32,33].

## Supporting information

S1 Fig Empirical cumulative distribution function plots of *Trichuris suis* EPG at each time point (EPG Week 6–14) and mean EPG throughout time course (EPG mean) for low weight gain animals (red) compared to high weight gain animals (blue) grouped by pig weight at week 0–7 (n = 174). p-values based on Wilcoxon signed rank tests are given in addition to the same p-values adjusted according to Holm’s method (Holm, 1979).
(TIF)

S2 Fig Empirical cumulative distribution function plots of *Trichuris suis* EPG at each time point (EPG Week 6–14) and mean EPG throughout time course (EPG mean) for low weight gain animals (red) compared to high weight gain animals (blue) grouped by pig weight at week 7–14 (n = 174). p-values based on Wilcoxon signed rank tests are given in addition to the same p-values adjusted according to Holm’s method (Holm, 1979).
(TIF)

S3 Fig Empirical cumulative distribution function plots of *Ascaris suum* EPG at each time point (EPG Week 6–14), mean EPG throughout time course (EPG mean) and worm counts for low weight gain animals (red) compared to high weight gain animals (blue) grouped by pig weight at weeks 0–7 (n = 174). p-values based on Wilcoxon signed rank tests are given in addition to the same p-values adjusted according to Holm’s method (Holm, 1979).
(TIF)

S4 Fig Empirical cumulative distribution function plots of *Ascaris suum* EPG at each time point (EPG Week 6–14), mean EPG throughout time course (EPG mean) and worm counts for low weight gain animals (red) compared to high weight gain animals (blue) grouped by pig weight at weeks 7–14 (n = 174). p-values based on Wilcoxon signed rank tests are given in addition to the same p-values adjusted according to Holm’s method (Holm, 1979).
(TIF)

S5 Fig Empirical cumulative distribution function plots of *Trichuris suis* EPG at each time point (EPG Week 6–14) and mean EPG throughout time course (EPG mean) for low weight gain animals (red) compared to high weight gain animals (blue) grouped by pig weight at week 0 (n = 174). p-values based on Wilcoxon signed rank tests are given in addition to the same p-values adjusted according to Holm’s method (Holm, 1979).
(TIF)

S6 Fig Empirical cumulative distribution function plots of *Ascaris suum* EPG at each time point (EPG Week 6–14), mean EPG throughout time course (EPG mean) and worm counts for low weight gain animals (red) compared to high weight gain animals (blue) grouped by pig weight at week 0 (n = 174). p-values based on Wilcoxon signed rank tests are given in addition to the same p-values adjusted according to Holm’s method (Holm, 1979).
(TIF)
