# Supplementary figures and images for: Assessing the impact of Ascariasis and Trichuriasis on weight gain using a porcine model
Source: PLoS Negl Trop Dis. 2022 Aug 19;16(8):e0010709. doi: 10.1371/journal.pntd.0010709 (PMC9390923; doi:10.1371/journal.pntd.0010709)

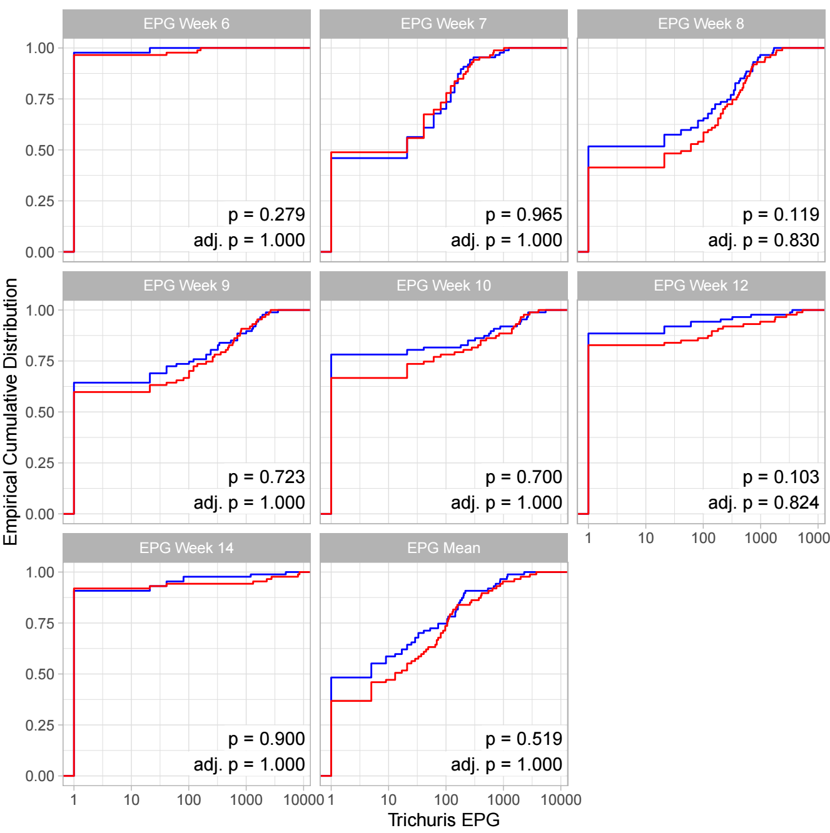

Supplement: S1 Fig — p-values based on Wilcoxon signed rank tests are given in addition to the same p-values adjusted according to Holm’s method (Holm, 1979). (TIF) [file pntd.0010709.s001.tif]

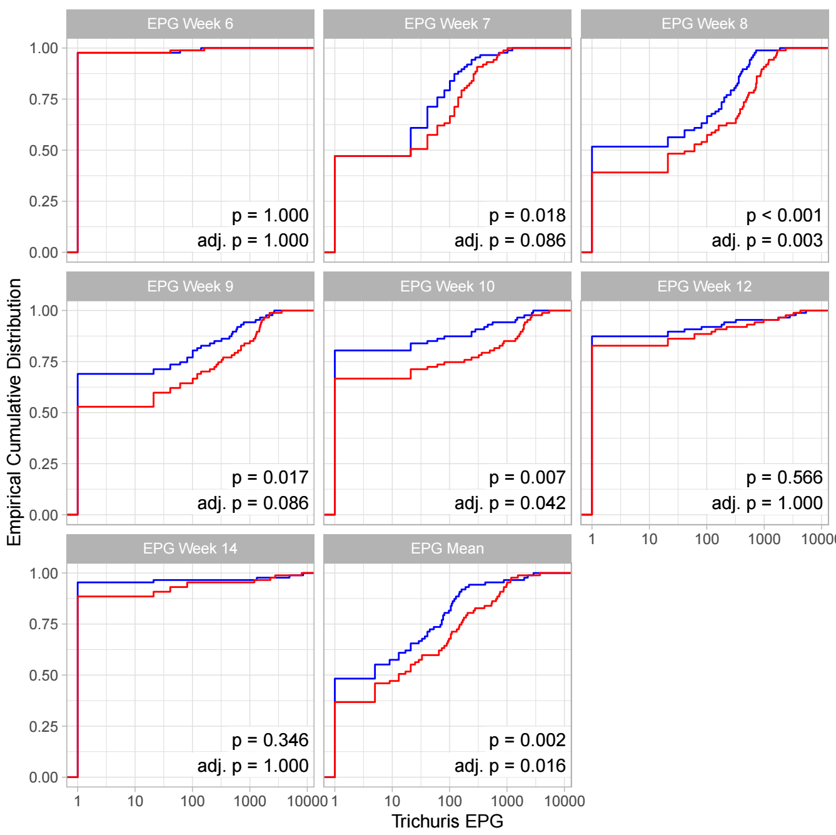

Supplement: S2 Fig — p-values based on Wilcoxon signed rank tests are given in addition to the same p-values adjusted according to Holm’s method (Holm, 1979). (TIF) [file pntd.0010709.s002.tif]

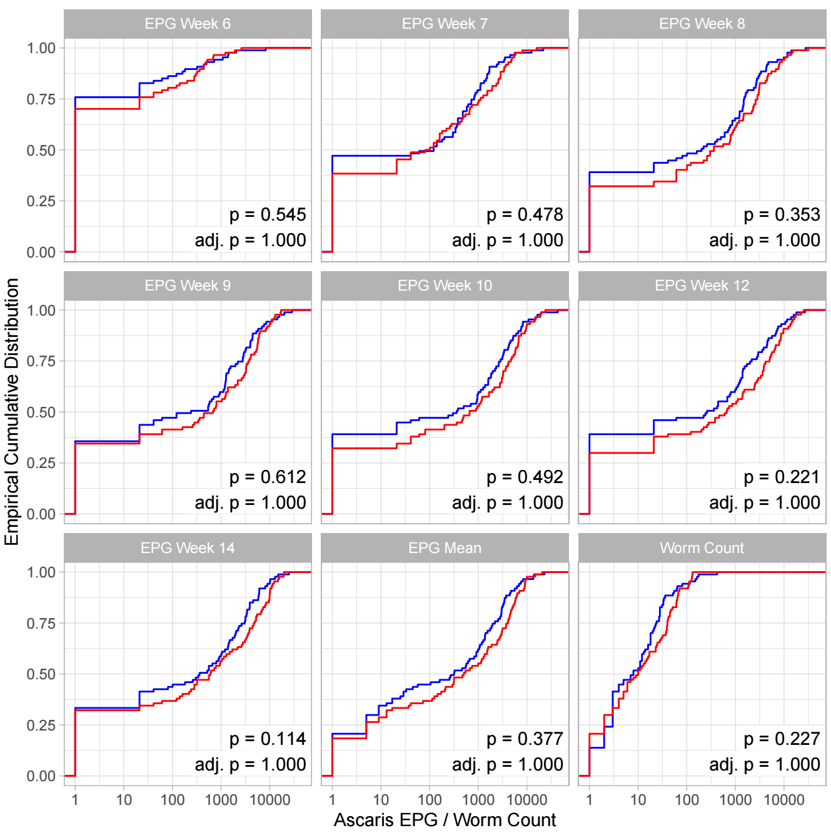

Supplement: S3 Fig — p-values based on Wilcoxon signed rank tests are given in addition to the same p-values adjusted according to Holm’s method (Holm, 1979). (TIF) [file pntd.0010709.s003.tif]

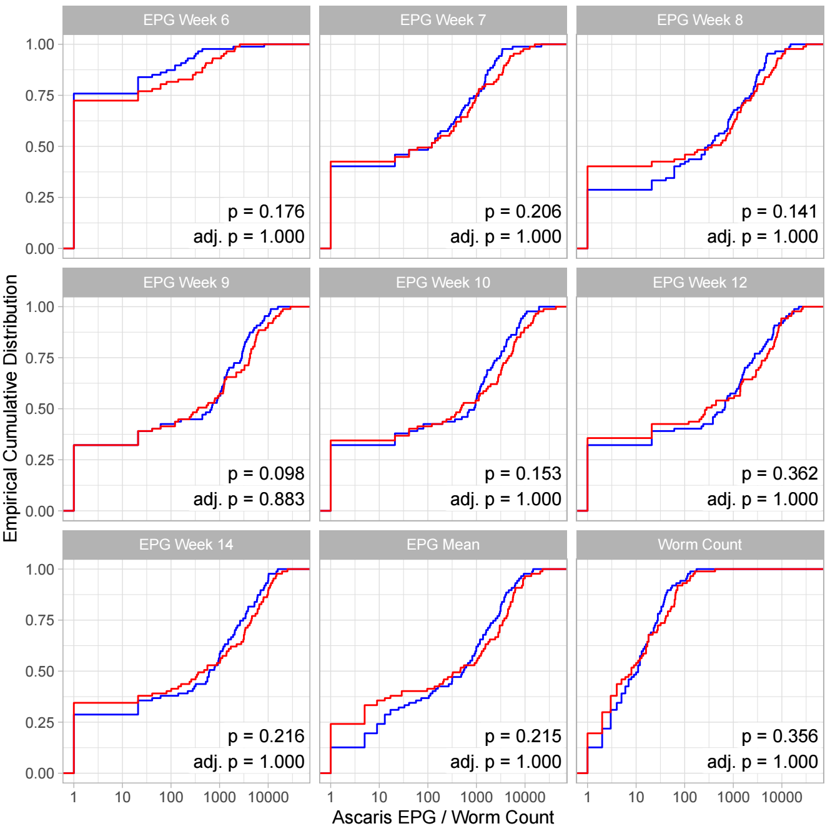

Supplement: S4 Fig — p-values based on Wilcoxon signed rank tests are given in addition to the same p-values adjusted according to Holm’s method (Holm, 1979). (TIF) [file pntd.0010709.s004.tif]

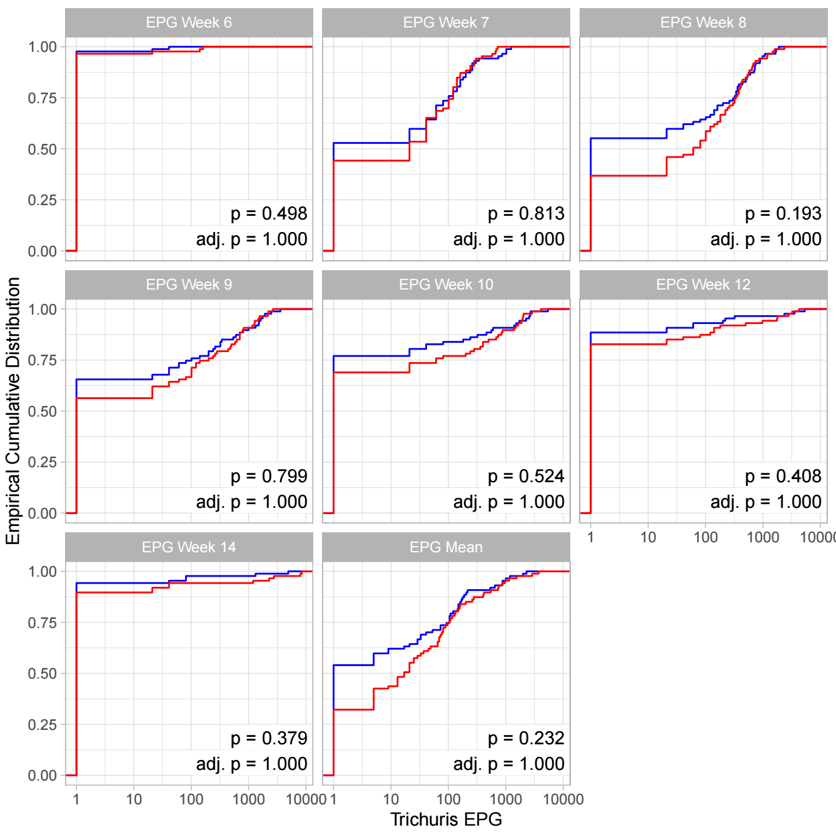

Supplement: S5 Fig — p-values based on Wilcoxon signed rank tests are given in addition to the same p-values adjusted according to Holm’s method (Holm, 1979). (TIF) [file pntd.0010709.s005.tif]

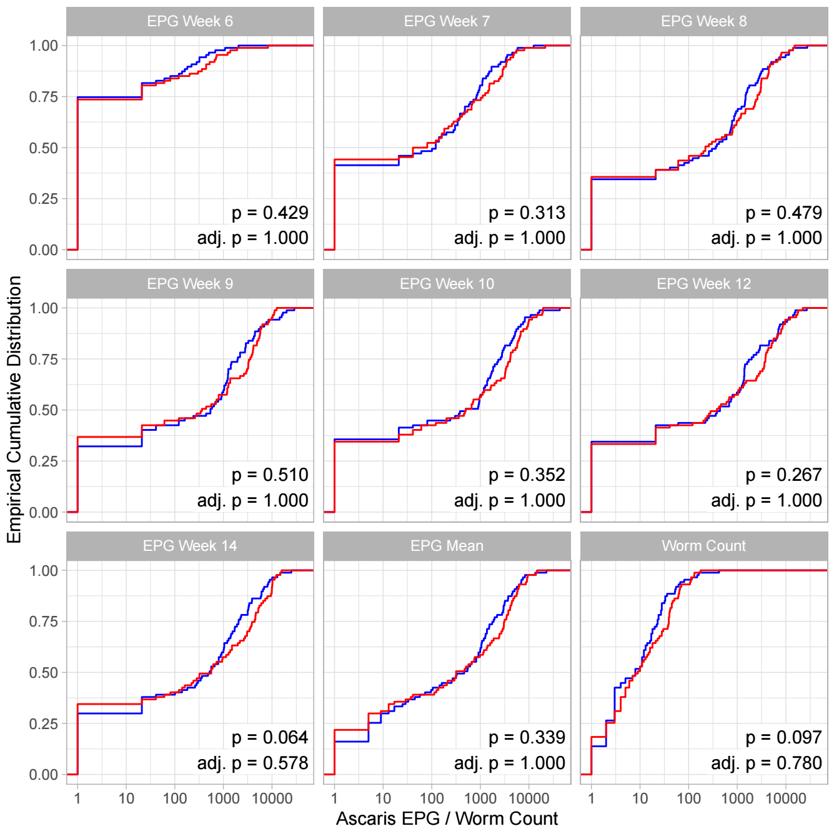

Supplement: S6 Fig — p-values based on Wilcoxon signed rank tests are given in addition to the same p-values adjusted according to Holm’s method (Holm, 1979). (TIF) [file pntd.0010709.s006.tif]
